# Novel MOF-Based Photocatalyst AgBr/AgCl@ZIF-8 with Enhanced Photocatalytic Degradation and Antibacterial Properties

**DOI:** 10.3390/nano12111946

**Published:** 2022-06-06

**Authors:** Ning Liu, Jie Zhang, Yanhua Wang, Qingjun Zhu, Xuan Zhang, Jizhou Duan, Baorong Hou

**Affiliations:** 1 Key Laboratory of Marine Environmental Corrosion and Bio-Fouling, Institute of Oceanology, Chinese Academy of Sciences, Qingdao 266071, China; ningouc@163.com (N.L.); zhuqingjun@qdio.ac.cn (Q.Z.); duanjz@qdio.ac.cn (J.D.); baoronghou@163.com (B.H.); 2 Key Laboratory of Marine Chemistry Theory and Technology, Ministry of Education, College of Chemistry and Chemical Engineering, Ocean University of China, Qingdao 266100, China; xuan20020136@163.com; 3 Open Studio for Marine Corrosion and Protection, Pilot National Laboratory for Marine Science and Technology (Qingdao), 1 Wenhai Road, Qingdao 266237, China

**Keywords:** photocatalysis, metal–organic frameworks, photocatalytic antibacterial, organic pollutants

## Abstract

A novel visible light-driven AgBr/AgCl@ZIF-8 catalyst was synthesized by a simple and rapid method. The composition and structure of the photocatalyst were characterized by XRD, SEM, UV-DRS, and XPS. It could be observed that the 2-methylimidazole zinc salt (ZIF-8) exhibited the rhombic dodecahedron morphology with the AgCl and AgBr particles evenly distributed around it. The composite photocatalyst AgBr/AgCl@ZIF-8 showed good photocatalytic degradation and antibacterial properties. The degradation rate of RhB solution was 98%, with 60 min of irradiation of visible light, and almost all *P. aeruginosaudomonas aeruginosa* (*P. aeruginosa*), *Staphylococcus aureus* (*S. aureus*), and *Escherichia coli* (*E. coli*) were inactivated under the irradiation of 90 min. In addition, the prepared catalyst had excellent stability and reusability. Based on the free radical capture experiment, ·O_2_^−^ and h^+^ were believed to be the main active substances, and possible photocatalytic degradation and sterilization mechanisms of AgBr/AgCl@ZIF-8 were proposed.

## 1. Introduction

Bacterial infection is a serious threat to human life and has attracted wide attention [1,2]. Among various antibacterial methods, photocatalytic sterilization technology has become the focus of people’s attention because of its good sterilization performance and quality of no secondary pollution [3]. As early as 1985, Matsunaga et al., discovered that TiO_2_ had a certain photocatalytic sterilization performance under ultraviolet irradiation [4]. Since then, research on photocatalytic sterilization materials and their sterilization mechanism has been widely reported [5,6,7]. In single-phase crystals, the separation rate of the electron-hole is slow and the recombination is easy, which seriously reduces the actual efficiency of the photocatalyst. Semiconductor doping is considered the most effective modification method, which can greatly improve the photocatalytic performance at a low cost, and has become a hot topic in recent years [8,9].

Metal–organic frameworks (MOFs) are porous coordination polymers with a micro/mesoporous structure [10,11]. Due to their larger specific surface area, adjustable pore diameter, controlled ion release, and other excellent properties, they have received extensive attention and are considered as the third generation of antibacterial agents [12,13]. The zeolitic imidazolate framework-8 (ZIF-8), formed by the reaction of Zn^2+^ with 2-methylimidazole, has been applied in the field of photocatalysis due to its high specific surface area, stable structure, and regular pores [14,15,16,17,18]. However, most MOF materials have a large band gap and cannot produce enough ROS for photocatalytic antibacterial application [19]. Therefore, the combination of ZIF-8 with other semiconductor catalysts is an effective way to improve its photocatalytic ability [14]. To date, a variety of composite photocatalysts have been synthesized. Fan et al., successfully prepared Ag/AgCl@ZIF-8 by doping Ag/AgCl onto ZIF-8 and the composite Ag/AgCl@ZIF-8 exhibited enhanced photocatalytic degradation of methylene blue [8]. Wang et al., synthesized a novel ZIF-8 modified MnFe_2_O_4_ magnetic catalyst by the one step method; the Zn–O–Fe structure was formed in the composite material due to the addition of ZIF-8. The composite exhibited enhanced light absorption properties and excellent photo-generated carrier generation and separation ability [20]. Yuan et al., prepared the core-shelled g-C_3_N_4_@ZIF-8 photocatalyst and the results showed that g-C_3_N_4_ could produce interfacial band bending, which could effectively promote the transfer of electrons to the surface of ZIF-8 [17]. Zeng et al., prepared the core-shell structure of the CdS@ZIF-8 composite, which exhibited enhanced photocatalytic performance compared with pure CdS NPs [18].

Silver halides (AgX, X = Cl, Br, and I) are common photosensitive materials and often used to modify photocatalysts to increase visible light absorption and improve overall photocatalytic ability [21]. In addition, the SPR effect of silver nanoparticles can enhance the separation ability of the electron-hole pair and improve the photocatalytic activity [22].

AgBr and AgCl have received extensive attention due to their excellent photocatalytic activity. However, the photocatalytic performance can be greatly reduced due to their serious aggregation, poor adsorption performance, fast electron-hole recombination rate, and poor stability. In published articles, AgCl was combined with ZIF-8 to prepare the composite photocatalyst. Additionally, there are few studies that combined AgCl, AgBr, and ZIF-8 to prepare composite catalysts and test the photocatalytic performance. The combination of silver-based semiconductors with MOFs is beneficial to overcome their shortcomings enhance their advantages, and further enhance the photocatalytic performance.

Therefore, in this paper, the MOF-based photocatalyst AgBr/AgCl@ZIF-8 was synthesized by a simple and rapid method. Then, the composition, structure, and photocatalytic properties of the photocatalyst were examined. The photocatalytic performance of the catalyst was tested by degrading the RhB solution and photocatalytic antibacterial experiment. Finally, the possible photocatalysis mechanism of the prepared photocatalyst was put forward according to ROS quenching experiment.

## 2. Experimental Section

### 2.1. Preparation of AgBr/AgCl@ZIF-8

AgCl@ZIF-8 was synthesized based on the previous article by a modified method [23,24]. In brief, 0.136 g of ZnCl_2_ and 0.657 g of 2-methylimidazole were put into 20 mL of methanol solution. Then, the two solutions were mixed and stirred for 30 min. Similarly, after 0.17 g of AgNO_3_ was completely dispersed in 10 mL of methanol solution, added it to the above combination and stirred overnight under dark conditions. Then, the solution was centrifuged, washed with ethanol, and dried at 60 °C for 6 h.

**Figure 1:**
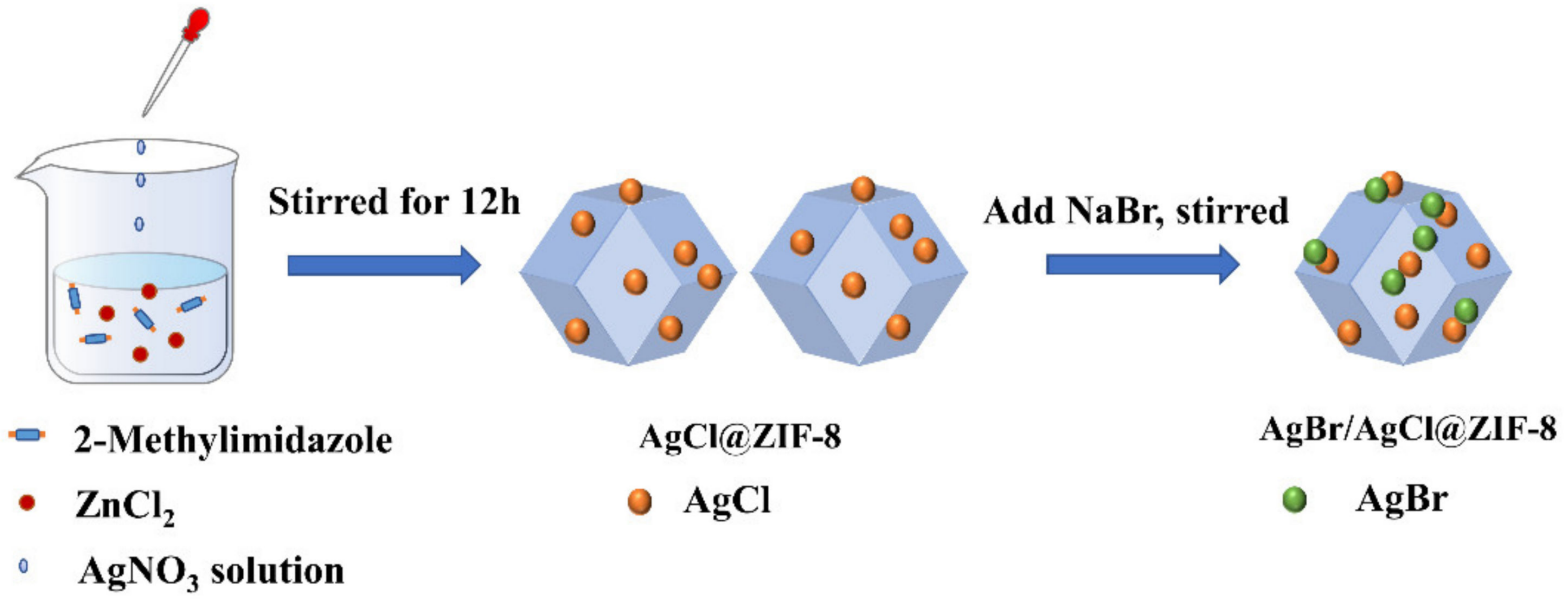
Flow chart of the preparation of AgBr/AgCl@ZIF-8.

In total, 0.1 g of AgCl@ZIF-8 was poured into 25 mL of deionized water and ultrasonic treatment was performed for 30 min to disperse it into homogeneous solution A. In total, 0.03 g of NaBr was poured into 25 mL of deionized water to obtain solution B, which was slowly dropped into solution A. Then, the pH value of the solution was adjusted to 7 and stirred overnight at room temperature in the dark. Finally, the reaction product was filtered and washed thoroughly with deionized water and ethanol, and dried overnight at 60 °C. This product was indicated as “AB/AC@Z3”. According to this method, the samples added with 0.01 g of NaBr and 0.05 g of NaBr were indicated as “AB/AC@Z1” and “AB/AC@Z5”. Figure 1 shows the specific synthesis process.

### 2.2. Characterization

The crystal structure of the sample was examined by XRD (Rigaku D/max-3C, Tokyo, Japan). The microstructures of the photocatalysts were characterized using SEM (Hitachi S-4800, Tokyo, Japan). The characteristics of the absorption spectra were detected using an ultraviolet-visible spectrophotometer (U-2900, Tokyo, Japan), during which ultrapure water was used as a reference.

### 2.3. Photocatalytic Performance

The photocatalytic properties of the nanoparticles were tested by decomposition of the RhB solution in visible light using a 350 W Xe lamp (XPA-7, Xujiang Electromechanical Plant, Nanjing, China), which was equipped with a 420 nm filter to ensure the output wavelength was 420–800 nm. First, 25 mg of material was added into a quartz tube containing 50 mL of 10 mg/L RhB solution, which was placed in a photocatalytic reactor and stirred for 30 min under dark conditions to realize the adsorption–desorption equilibrium. Then, the light was turned on and the samples were taken out and filtered at regular intervals. By detecting the absorbance of the supernatant, the amount of the remaining RhB was indicated until it was completely degraded. The degradation rate was calculated according to the following: degradation efficiency (%) = (1 − C_t_/C_0_) × 100, where t represents the irradiation time and C_0_ and C_t_ are the concentrations of RhB after illumination for 0 min and t minutes [25].

In this paper, *P. aeruginosa*, *E. coli*, and *S. aureus* were chosen to evaluate the photocatalytic antifouling ability of the synthetic materials. Typically, 25 mg of the catalyst and 49 mL of PBS were added into quartz tube. Then, it was put into the photochemical reactor and stirred for 30 min. Later, 1 mL of bacterial suspension was added for the photocatalytic antibacterial experiment and then the light was switched on. A fixed amount of bacterial solution was extracted every 30 min during the experiment and diluted with PBS solution by the decimal method. In total, 100 μL of suspension with different dilution times was evenly coated on LB agar plates and then placed in a constant temperature incubator culture at 37 °C for 24 h as well as counted. Each group was measured three times in parallel.

### 2.4. Free Radical Scavenging Experiment

The free radical scavenging experiment can determine the main active species that play a major role in the photocatalytic experiment. In total, 1 mmol of 1,4-benzoquinone (BQ, scavengers of O_2_^−^), Isopropyl alcohol (IPA, scavengers of·OH), and sodium oxalate (MSDS, scavengers of h^+^) were selected as scavengers to be added to the photocatalytic degradation process. The main active species in the photocatalytic process can be inferred from the alteration of photocatalytic degradation properties [3].

## 3. Results and Discussion

### 3.1. The Structure of Photocatalysts

**Figure 2:**
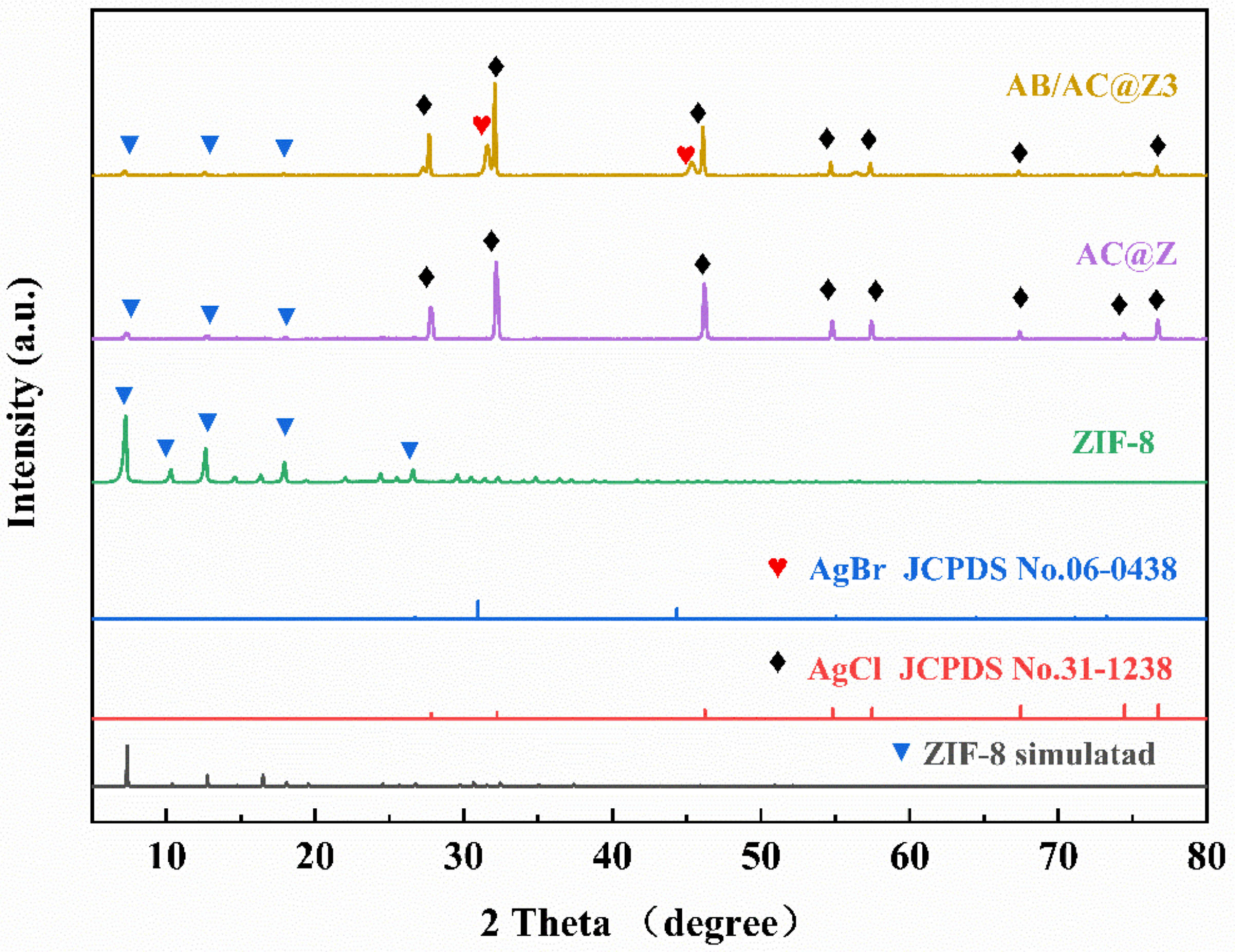
The X-ray diffraction patterns of the prepared photocatalysts.

Figure 2 shows the X-ray diffraction patterns of the prepared materials. The peak positions of ZIF-8 were in accordance with the standard peak [26]. For AC@Z, the diffraction peaks at 2θ = 27.34°, 10.36°, 12.72°, 16.31°, and 18.02° were attributed to the diffraction peak of ZIF-8. In addition, the obvious peaks that appeared at 27.78°, 32.23°, 46.20°, 54.79°, 57.45°, 67.45°, 74.39°, and 76.73° belonged to the (111), (200), (220), (311), (222), (400), (331), and (420) planes of AgCl [27]. The XRD pattern of AB/AC@Z with different proportions retained strong diffraction peaks of ZIF-8 and AgCl. In addition, the diffraction peaks at 2θ = 26.84°, 30.99°, and 45.28° were assigned to the (111), (200), and (220) planes of AgBr [28,29]. With the addition of AgBr and AgCl, obvious AgBr and AgCl diffraction peaks appeared and the intensity of the diffraction peak of ZIF-8 was weakened, which indicates that AgBr, AgCl, and ZIF-8 were successfully doped. There were no other obvious impurity peaks in the X-ray diffraction patterns of the composite material, which indicates that the synthesized catalyst had high purity and high crystallinity.

### 3.2. The Morphologies of the Photocatalysts

**Figure 3:**
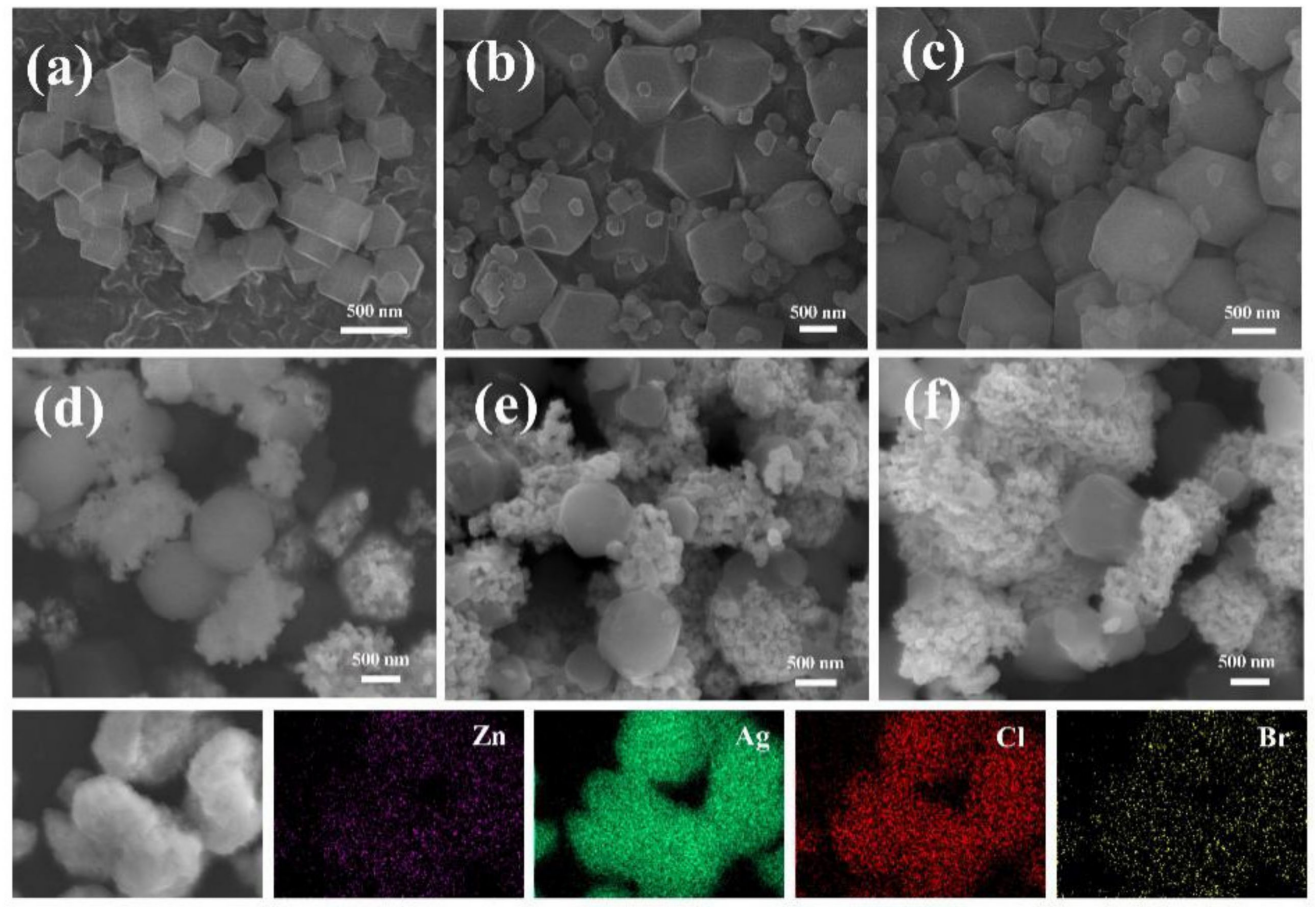
SEM images of (**a**) ZIF-8, (**b**,**c**) AC@Z, and (**d**–**f**) AB/AC@ZX (X = 1, 3, and 5).

The morphology of ZIF-8, AC@Z, and the different molar ratios of AB/AC@Z were observed by SEM. As shown in Figure 3a, ZIF-8 exhibited a rhombic dodecahedron structure with a diameter of about 350 nm. Figure 3b,c show the morphology of AC@Z and AgCl particles of approximately 200 nm in diameter uniformly embedded around ZIF-8. Figure 3d–f show the morphologies of AB/AC@ZX (X = 1, 3, and 5) nanoparticles. It could be seen that many particles were evenly distributed around ZIF-8, which might be due to the partial conversion of AgCl to AgBr. Furthermore, with the increase of the addition of NaBr, more small particles were produced. When the loading amount was the largest, the nanoparticles began to accumulate. The following are the elemental maps of AB/AC@Z. The Zn, Ag, Cl, and Br elements were distributed regularly, which further proves the successful synthesis of the material.

### 3.3. The Surface Elemental Compositions

**Figure 4:**
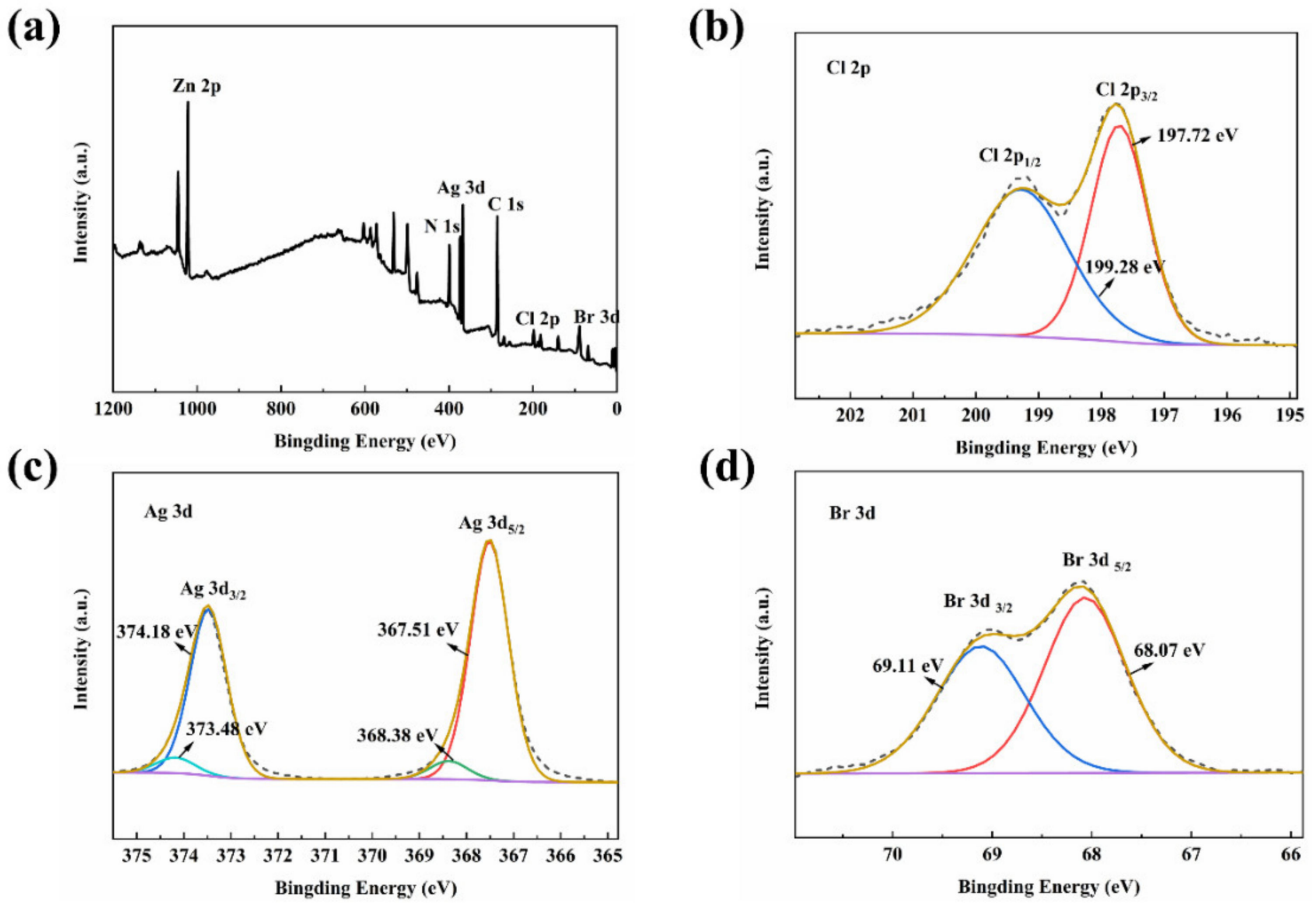
XPS spectra of AgBr/AgCl@Z3 composition: (**a**) survey, (**b**) Cl 1s, (**c**) Ag 3d, and (**d**) Br 2p.

The electronic properties of the photocatalyst were further tested by XPS. As shown in Figure 4a, the Zn, N, Ag, C, Cl, and Br elements were shown in the full spectrum of XPS. The Cl 2p signal is displayed in Figure 4b, in which the peaks at 197.72 eV and 199.28 eV belong to Cl 2p_1/2_ and Cl 2p_3/2_, respectively [30]. In the Ag 3d XPS spectra, the Ag 3 d peaks could be divided into four peaks, of which the strong peaks located at 367.51 eV and 374.18 eV belong to Ag^+^, and the other two weak peaks belong to metal Ag [3,30]. The appearance of Ag could be attributed to the reduction of Ag^+^ during the reaction process. Figure 4d displays the Br 3d level spectrum. The Br 3d_5/2_ and Br 3d_3/2_ spinorbital photoelectrons were located at 68.07 eV and 69.11 eV, respectively [31,32]. In addition, no other obvious impurity peaks were observed in the XPS chromatogram, which indicates that the synthesized material was composed of C, N, Cl, Zn, and Ag elements.

### 3.4. The Photocatalytic Activity

**Figure 5:**
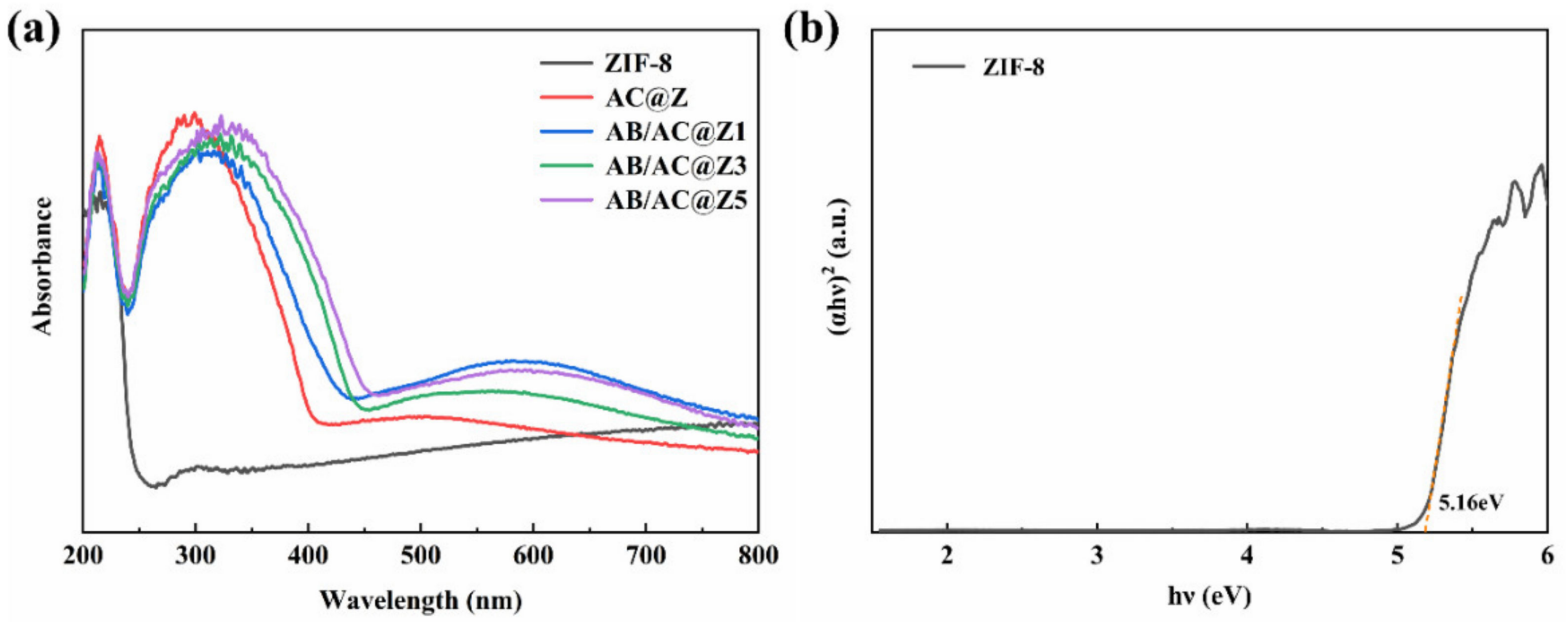
(**a**) The UV-DRS for the photocatalytic materials. (**b**) Plots of (αh*ν*) vs. h*ν* for ZIF-8.

The energy band structure of the photocatalyst was closely related to its performance. Figure 5a shows the UV-DRS spectrum of the prepared sample. According to the spectrum, ZIF-8 had absorption only in the ultraviolet region and the absorption edge was about 250 nm, while AC@Z had obvious absorption due to the addition of AgCl [33]. The AB/AC@Z composite exhibited excellent light absorption properties, indicating that the composite catalyst greatly expanded the light absorption range and enhanced the light absorption capacity. According to Tauc plots ((αh*ν*)^2^ = A(h*ν* − Eg); the calculated band gap value of ZIF-8 was 5.10 Ev (Figure 5b), which is consistent with those previously studied [34]. Due to the doping of AgCl and AgBr, the absorption range of AB/AC@Z was significantly increased. The successful doping of the semiconductor materials played a synergistic role and the composite material exhibited excellent photophysical properties.

**Figure 6:**
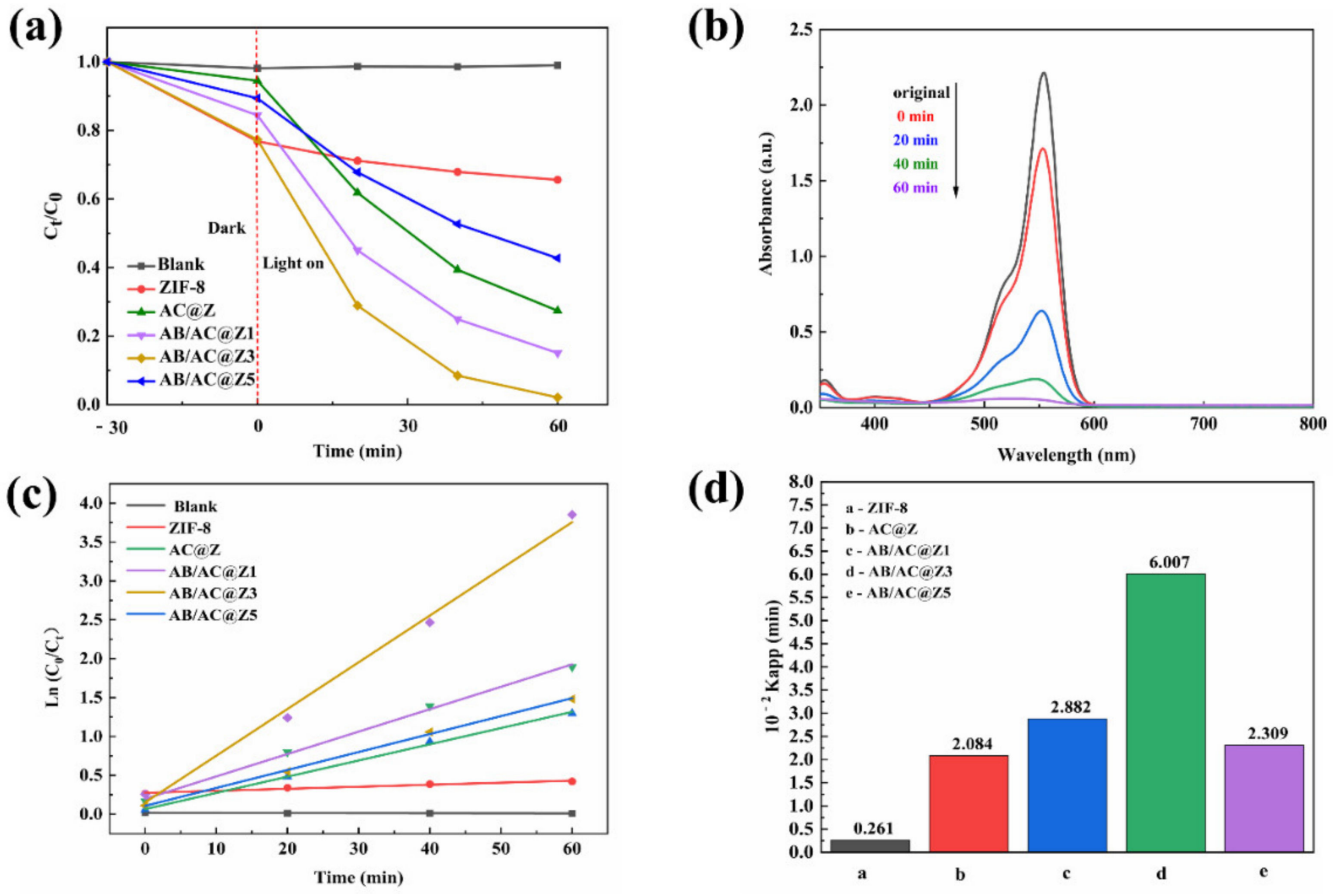
(**a**) The photodegradation of RhB with different materials. (**b**) UV-DRS of RhB solutions treated with AB/AC@Z3. (**c**,**d**) The first-order kinetic constants of different materials.

The photocatalytic performance of the synthesized materials was assessed by the decomposition of RhB under the irradiation of visible light. In order to reduce the impact of adsorption and light on RhB, adsorption equilibrium experiments under dark conditions and blank control experiments were performed. As shown in Figure S1, the concentration of RhB showed no significant changes after 30 min of dark reaction, indicating that the adsorption equilibrium was reached. After that, the light source was turned on for the photocatalytic degradation experiment. The blank experiment was carried out under the condition of light and catalyst-free. As can be seen in Figure 6a, the degradation rate of RhB in the blank experiment was low, indicating that the light factor has little effect on the photodegradation effect of RhB and can be ignored [35]. At the same time, ZIF-8, AC@Z, and AB/AC@Z exhibited different degrees of degradation of RhB, among which AB/AC@Z3 showed the best degradation performance. As can be seen from Figure 6b, the absorption peak of the RhB solution at 554 nm gradually decreased with the increase of illumination time and the degradation amount of RhB reached 98% after 60 min of irradiation. In Figure 6c,d, the first-order reaction kinetic constant Kapp [36] of the degradation ability of the AB/AC@Z3 composite on RhB was the largest, which was 0.0601 min^−1^ and it was 23 times that of ZIF-8, indicating that the formation of the composite enhanced its photocatalytic performance.

### 3.5. Photocatalytic Antibacterial Efficiency

**Figure 7:**
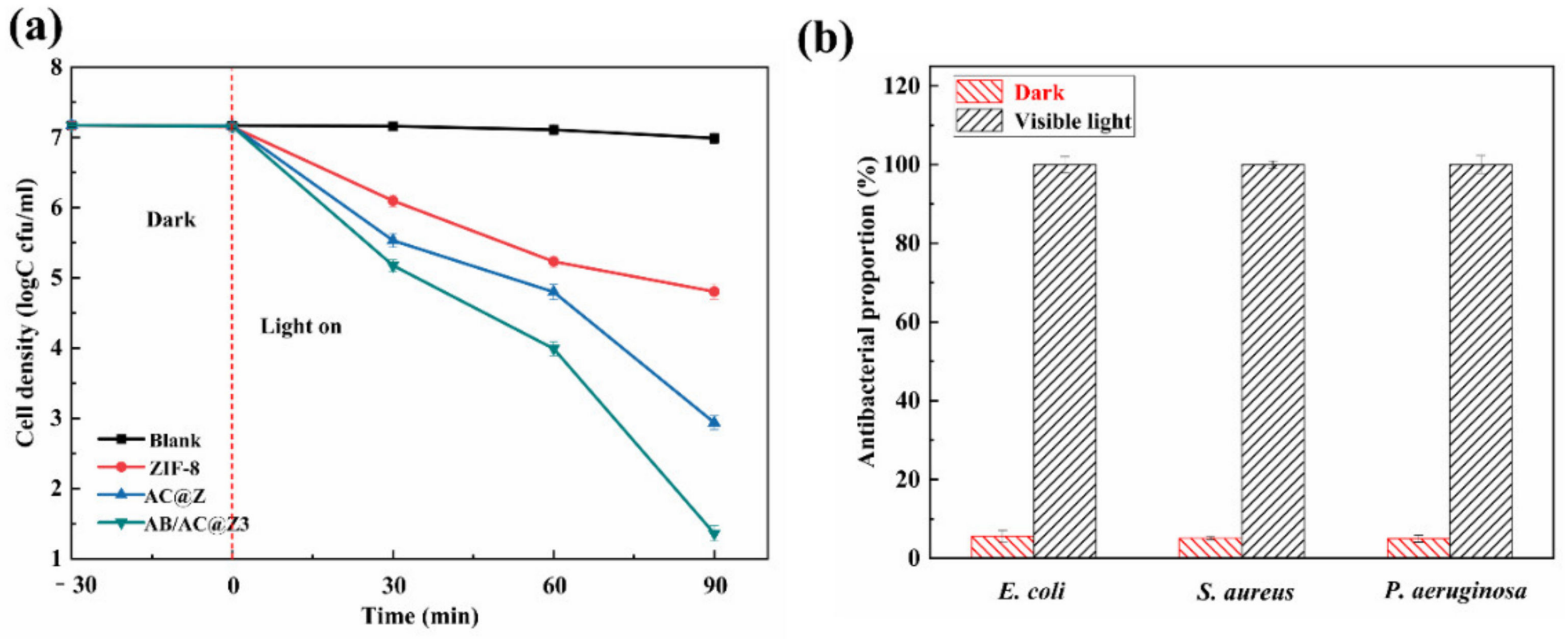
(**a**) Survival curves of *E. coli* treated with different catalysts under visible light. (**b**) Total percentage of inactivation of *E. coli*, *S. aureus*, and *P. aeruginosa* by AB/AC@Z3 under dark and light conditions.

In this paper, *E. coli*, *P. aeruginosa*, and *S. aureus* were used to measure the photocatalytic antibacterial properties of catalysts. The concentration of the catalyst used in the reaction was 0.5 mg/L and the illumination time was 90 min. Figure 7a shows the survival curve of *E. coli*. As can be seen from the blank experiment, the bacterial concentration hardly changed, which shows that the illumination and other environment factors had little effect on the growth of bacteria. After 90 min of illumination, AB/AC@Z3 had a better photocatalytic antibacterial effect than ZIF-8 and AC@Z, mainly because the combination of AgCl, AgBr, and ZIF-8 enhanced the overall photocatalytic capacity. As can be seen in Figure 7b, the catalyst had little effect on three kinds of bacteria under dark conditions, indicating that the photocatalyst is non-toxic to bacteria. Additionally, almost all bacteria were killed after 90 min of the photocatalytic reaction with the photocatalyst and the percentage of the inactivated bacteria to the total bacteria of *E. coli*, *S. aureus*, and *P. aeruginosa* reached 99.9998%, 99.9997%, and 99.9985%, respectively.

**Figure 8:**
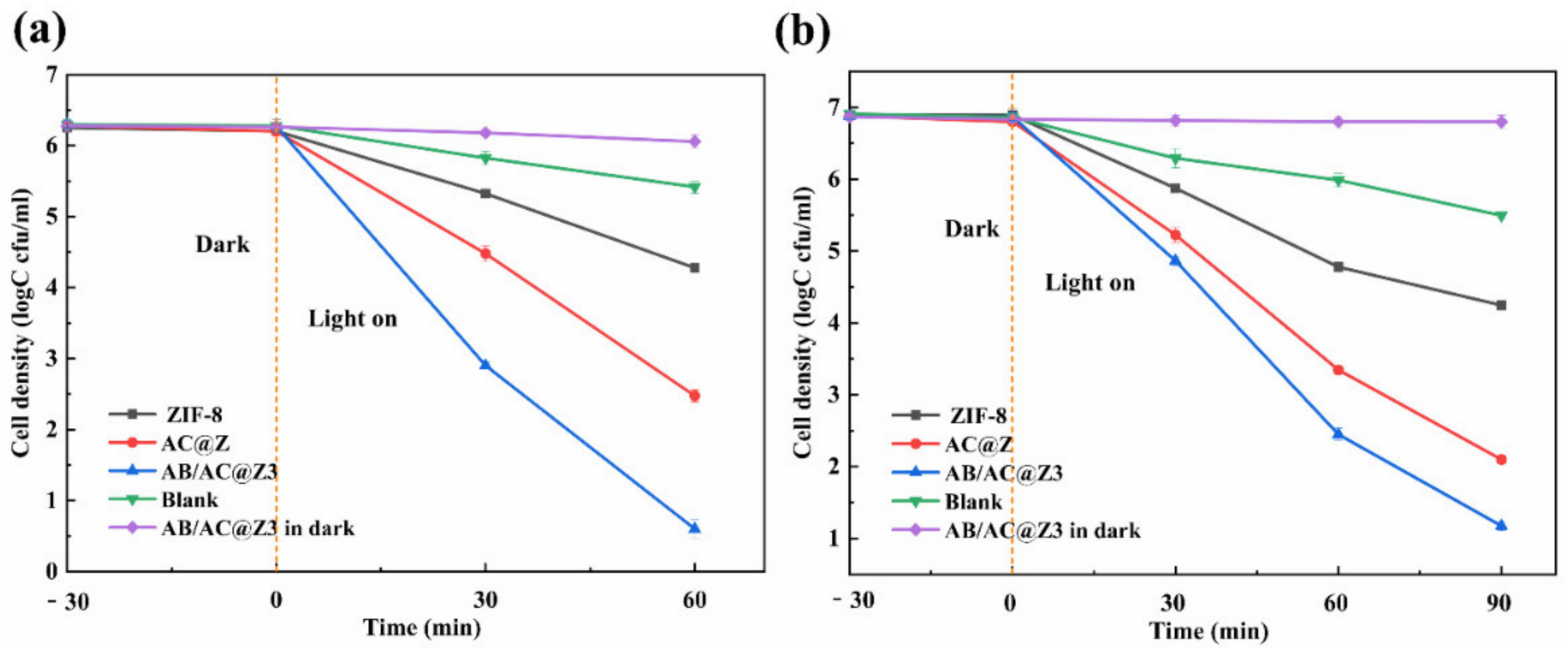
(**a**) Survival curves of *E. coli* treated with different catalysts under the simulated sunlight. (**b**) Survival curves of *P. aeruginosa* treated with different catalysts under the simulated sunlight.

Moreover, we removed the filter and carried out the photocatalytic antibacterial experiment under simulated sunlight. Both the species of bacteria and concentration of the catalyst were the same as those under visible light. Figure 8a shows the survival curve of *E. coli*. It could be seen from the blank experiment that the concentration of bacteria decreased slightly after 60 min, which may have been due to the irradiation of ultraviolet rays in the whole spectrum. In addition, ZIF-8 under the simulated sunlight showed a better antibacterial effect than that under visible light irradiation, which may have been due to its better light absorption capacity in the ultraviolet region, and AC@Z and AB/AC@Z3 also showed better antibacterial performance. Figure 8b shows the survival curve of *P. aeruginosa*. AB/AC@Z3 can inactivate almost all bacteria in under 90 min of the irradiation of the simulated sunlight. Finally, the percentage of the inactivated bacteria to the total bacteria of *E. coli*, *S. aureus* reached 99.9998% and 99.9996% within 60 min, while the percentage of the inactivated bacteria to the total bacteria of *P. aeruginosa* reached 99.9997% within 90 min. Compared with ZIF-8 and AB/AC@Z, the prepared composite material had a better photocatalytic antibacterial effect.

### 3.6. Stability and Reusability

**Figure 9:**
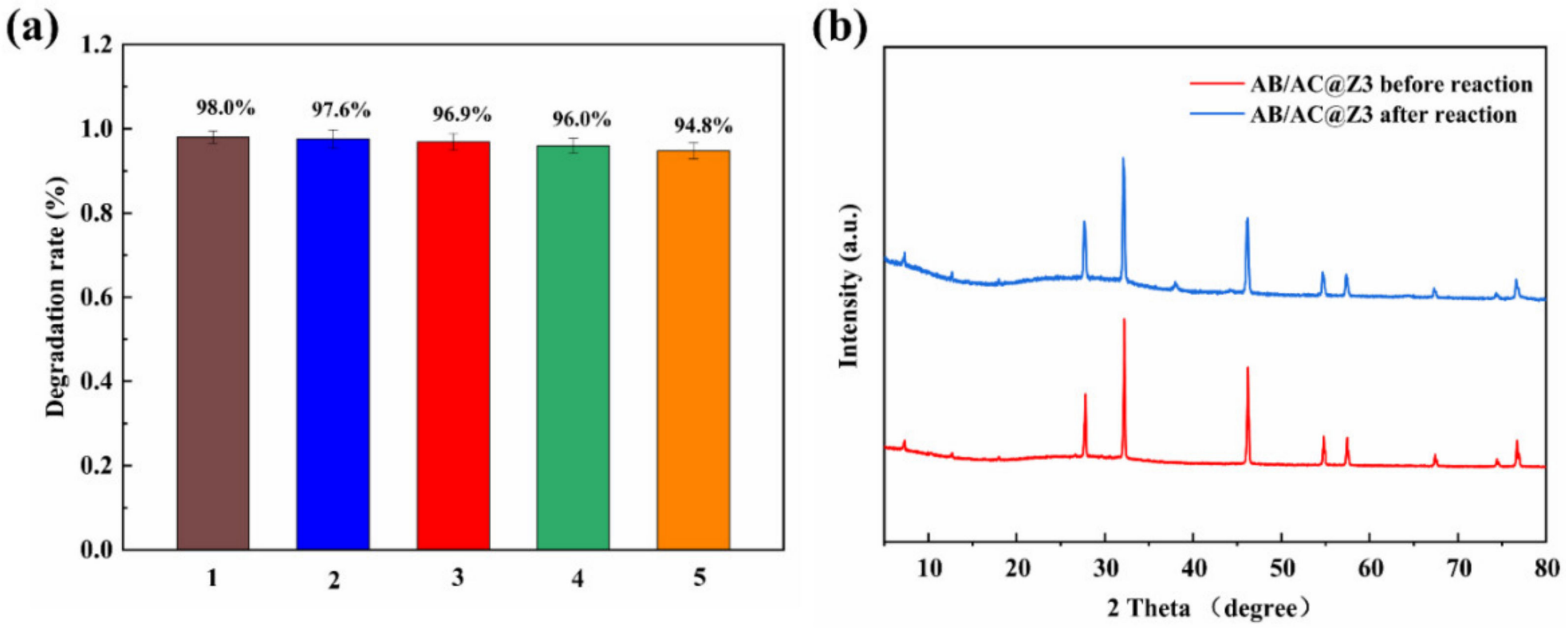
(**a**) Cyclic experiment of degradation of RhB solution. (**b**) XRD patterns of catalyst after recycling experiments.

In order to test the stability and reusability of the synthesized materials, the photocatalyst was used repeatedly to degrade RhB. After each degradation, the catalyst was cleaned and dried thoroughly for the next photocatalytic degradation experiment. As can be seen from Figure 9a, after five photocatalytic reactions, the decomposition rate of the organic pollutants only decreased by 3.4%, which may have been due to the loss of material in each cleaning process. As show in Figure 9b, compared with the newly prepared catalyst, a new peak appeared at 2θ = 38° and the peak at 38.1° belongs to Ag according to previous studies. It can be deduced that part of Ag (I) had been reduced to Ag during the reaction. Furthermore, the photocatalytic performance of the material did not change significantly. Hence, the synthesized materials have good stability and reusability.

### 3.7. Photocatalytic Mechanism

**Figure 10:**
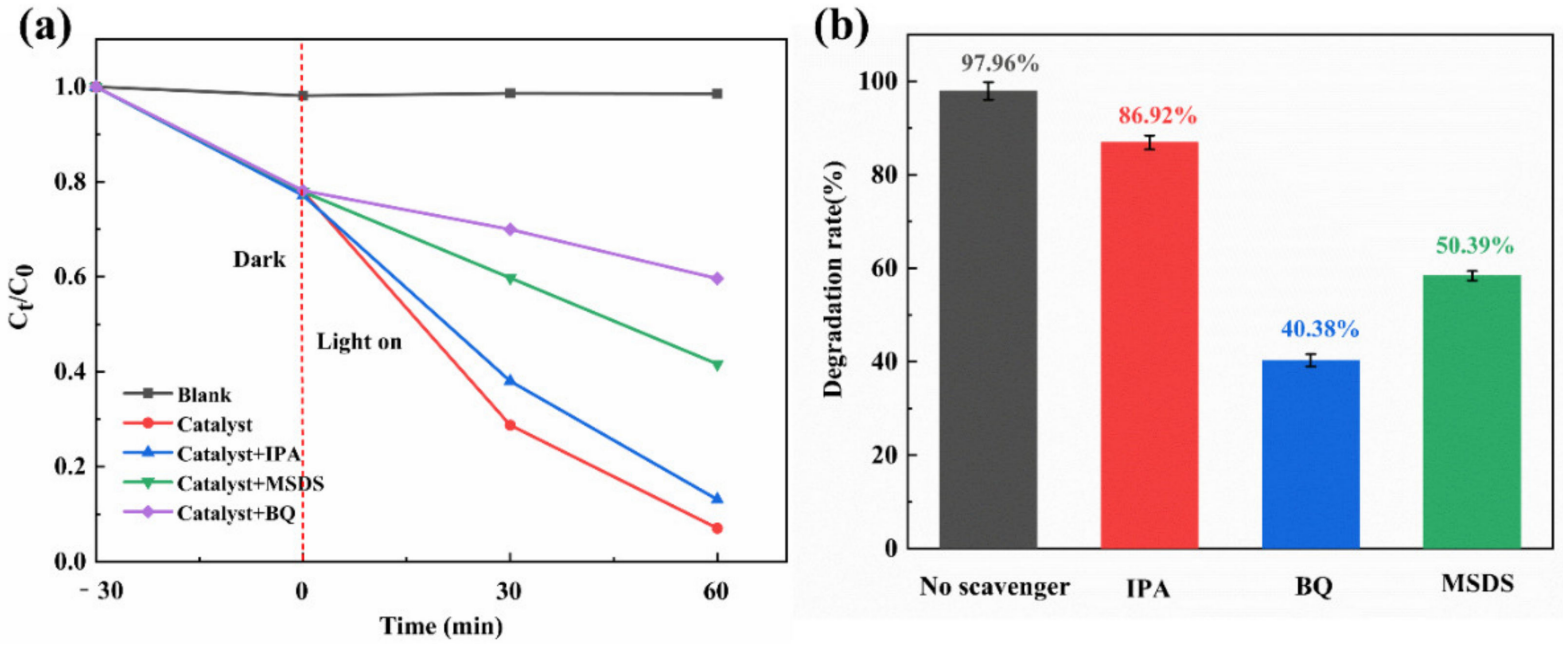
(**a**) Free radical scavenging experiment of AB/AC@Z3. (**b**) Degradation rate of RhB after adding different kinds of scavengers.

According to previous reports, free radical active species play an important role in the photocatalytic reaction [3]. Free radical scavenging experiments were conducted to verify the contribution of different active species in the photocatalytic reaction. As shown in Figure 10a,b, when exposed to visible light for 60 min without a scavenger, 98% of RhB can be degraded. When 1 mmol of IPA was added, the degradation effect was slightly weakened to 86.92%, suggesting that ·OH only plays minor roles in the reaction process [37]. When 1 mmol of BQ was added, the degradation rate was significantly reduced to 40.38% and the photocatalytic effect of the catalyst was inhibited. In addition, when 1 mmol of MSDS was added, only 50.39% of RhB could be degraded, indicating that ·O_2_^−^ and h^+^ are the primary active substances in this reaction. Additionally, we also tested the free radical trapping experiments’ antibacterial process (Figure S2) and all testers determined that h^+^ and ·O_2_^−^ were the main free radical active species in the reaction process.

**Scheme 1:**
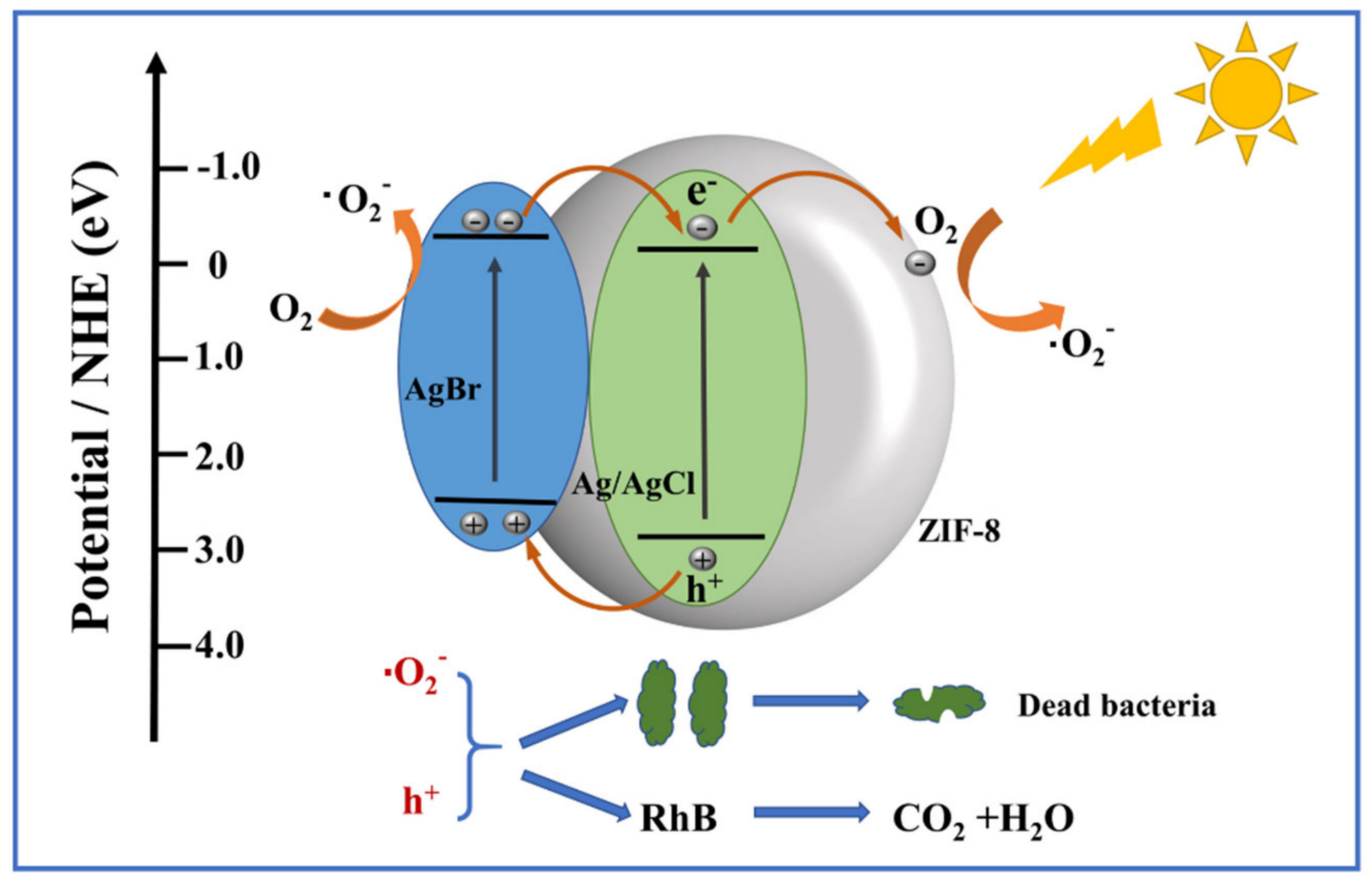
Possible mechanism of photocatalytic reaction in AB/AC@Z system.

A possible photocatalytic degradation and bactericidal mechanism of AB/AC@Z was proposed in combination with radical trapping experiments. According to previous studies, the conduction band (CB) of AgBr is −0.32 eV and the valence band (VB) is 2.58 eV, and the electrons on VB can be excited to CB under the irradiation of light [25]. During the photochemical reaction, part of Ag^+^ on AgCl will be reduced to Ag and Ag/AgCl, as a whole, will greatly increase the absorption performance of visible light. Studies have shown that the CB and VB values of Ag/AgCl are −0.16 eV and 2.93 eV, respectively [38]. So thatthe electrons on VB can be excited to its conduction band. The electrons on the CB of AgBr can be transferred to the CB of Ag/AgCl, while the holes on the VB of Ag/AgCl can be migrated to the VB of AgBr [39,40]. Due to the SPR effect of the Ag, electrons are transferred from Ag/AgCl to the CB of ZIF-8. As the CB position of ZIF-8 (−0.86 eV vs. NHE) is obviously more negative than that of E_0_ (O_2_/·O_2_^−^) [41], the photoelectrons generated by the above reaction are captured by a large amount of O_2_ adsorbed on the surface of ZIF-8 and then reacted with it to produce active oxygen (·O_2_^−^). Therefore, ZIF-8 plays the role of molecular oxygen activation in AB/AC@Z. At the same time, the ZIF-8 generates electron-hole pairs under the irradiation of light and the CB (LUMO) of ZIF-8 is injected with SPR electrons, which enhances the overall photocatalytic performance. Additionally, this structure can greatly reduce the recombination of electron-hole pairs. The electrons in the conduction band of AgBr can also react with O_2_ to generate ·O_2_^−^. The active substances ·O_2_^−^ and h^+^ can not only decompose the pollutant molecules into CO_2_ and H_2_O but it also can kill bacteria completely [42]. As shown in Scheme 1, when bacteria are exposed to the photocatalyst, the ·O_2_^−^ and h^+^ generated by the photocatalyst will actively attack the cell wall and cell membrane of bacteria, leading to cell membrane rupture and severe damage to the cytoplasm and deoxyribonucleic acid molecules [25,43]. Finally, the bacteria are almost completely inactivated. Therefore, the reaction process possibly involved in the photocatalytic reaction is shown in the following formula [44].

AgBr/AgCl@ZIF-8 + h*ν* → (e^−^ + h^+^) (1)

e^−^ + O_2_ →·O_2_^−^ (2)

organic pollutants (RhB) + ·O_2_^−^ + h^+^ → CO_2_+ H_2_O (3)

Live bacteria + ·O_2_^−^+ h^+^ → Dead bacteria (4)

## 4. Conclusions

In this paper, a novel AgBr/AgCl@ZIF-8 photocatalyst with different proportions was successfully synthesized via a simple and rapid method. The AgCl and AgBr nanoparticles were successfully embedded around ZIF-8. Due to the excellent light absorption properties of the composite photocatalyst and the large specific surface area of ZIF-8, a large number of electron-hole pairs can be generated and transferred rapidly to participate in the reaction, thus enhancing the photocatalytic performance. In the photocatalytic sterilization experiment, 99.998% of *E. coli*, *S. aureus*, and *P. aeruginosa* cells were completely inactivated after 90 min of irradiation of light. In addition, AB/AC@Z exhibited good stability in the cycling experiments. This research provides a new idea for the construction of MOF-based photocatalytic antibacterial materials with high performance.

## Data Availability

The data presented in this study are available upon request from the corresponding authors.

## Supplementary Materials

The supporting information can be downloaded at: https://www.mdpi.com/article/10.3390/nano12111946/s1. Table S1: The EDX analysis of AB/AC@Z3. Figure S1: Adsorption experiments of catalyst on RhB under dark conditions. Figure S2: Photocatalytic antibacterial proportion after adding scavenger. Figure S3. (a) Photocatalytic reaction instrument (b,c) Internal structure and principle of reaction box.
