# Supplementary material for: Novel MOF-Based Photocatalyst AgBr/AgCl@ZIF-8 with Enhanced Photocatalytic Degradation and Antibacterial Properties
Source: Nanomaterials (Basel). 2022 Jun 6;12(11):1946. doi: 10.3390/nano12111946 (PMC9182966; doi:10.3390/nano12111946)
Supplement: Supplementary file 1 [file nanomaterials-12-01946-s001.zip › nanomaterials-1752649-supplementary materials.pdf]

nanomaterials

Supplementary Materials

Novel MOF-Based Photocatalyst AgBr/AgCl@ZIF-8 with Enhanced Photocatalytic Degradation and Antibacterial Properties

Ning Liu [1,2,3], Jie Zhang [1,3,*], Yanhua Wang [2,*], Qingjun Zhu [1,3], Xuan Zhang [2], Jizhou Duan [1,3] and Baorong Hou [1,3]

[1] Key Laboratory of Marine Environmental Corrosion and Bio-Fouling, Institute of Oceanology, Chinese Academy of Sciences, Qingdao 266071, China; ningouc@163.com (N.L.); zhuqingjun@qdio.ac.cn (Q.Z.); duanjz@qdio.ac.cn (J.D.); baoronghou@163.com (B.H.)
[2] Key Laboratory of Marine Chemistry Theory and Technology, Ministry of Education, College of Chemistry and Chemical Engineering, Ocean University of China, Qingdao 266100, China; xuan20020136@163.com
[3] Open Studio for Marine Corrosion and Protection, Pilot National Laboratory for Marine Science and Technology (Qingdao), 1 Wenhai Road, Qingdao 266237, China
* Correspondence: zhangjie@qdio.ac.cn (J.Z.); wyhazz@163.com (Y.W.); Tel.: +86-532-82898851 (J.Z.); +86-13675425612 (Y.W.)

EDX analysis of AB/AC@Z. As shown in Table S1, C, N, Cl, Zn, Ag, Br in the composite AB/AC@Z3 were detected by EDX analysis, indicating that the composite was composed of AgCl, AgBr and ZIF-8. It can be noticed that the atomic ratio of Cl and Br was close to the value of 1:1. It is also inferred from the amount of Ag that Ag^{+} may be partially reduced to Ag. Th*e* XPS results also proved the existence of Ag. Therefore, SEM and EDX analysis confirmed that AgCl and AgBr successfully combined with ZIF-8.

Table S1. The EDX analysis of AB/AC@Z3.

Elt.	Line	Intensity (c/s)	Atomic %	Atomic Ratio	Conc.	Units
**C**	Ka	120.58	23.668	1.0000	4.602	wt.%
**N**	Ka	15.39	8.330	0.3519	1.889	wt.%
**Cl**	Ka	565.29	12.487	0.5276	7.166	wt.%
**Zn**	Ka	63.33	7.464	0.3154	7.901	wt.%
**Br**	Ka	5.56	12.068	0.5099	15.609	wt.%
**Ag**	La	1,242.31	35.984	1.5204	62.833	wt.%
			100.000		100.000	Wt.%

Adsorption experiment of RhB in dark condition. The adsorption experiments of the catalyst were performed under dark conditions to detect the time to reach adsorption equilibrium. As shown in Fig. S1, As the reaction progressed, the concentration of RhB began to decrease. When the reaction time reached 30 min, the concentration of RhB showed no significant change, indicating that adsorption equilibrium was reached after 30 min dark reaction.

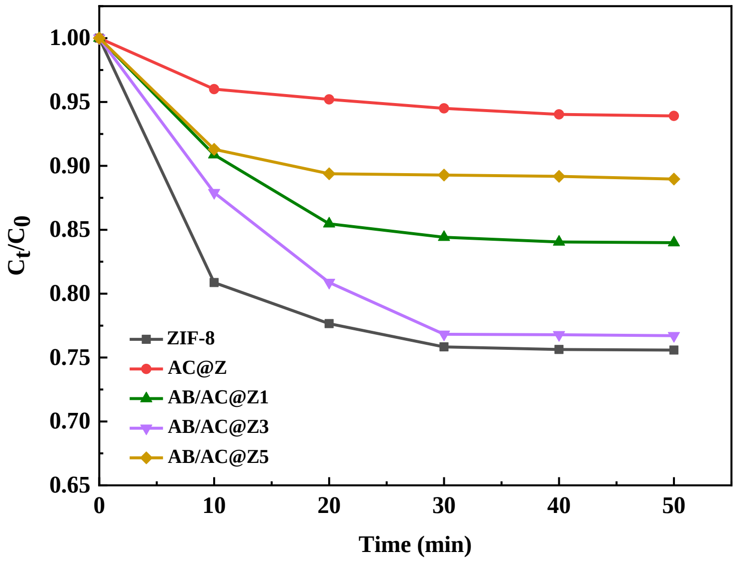

Figure S1. Adsorption experiments of catalyst on RhB under dark conditions.

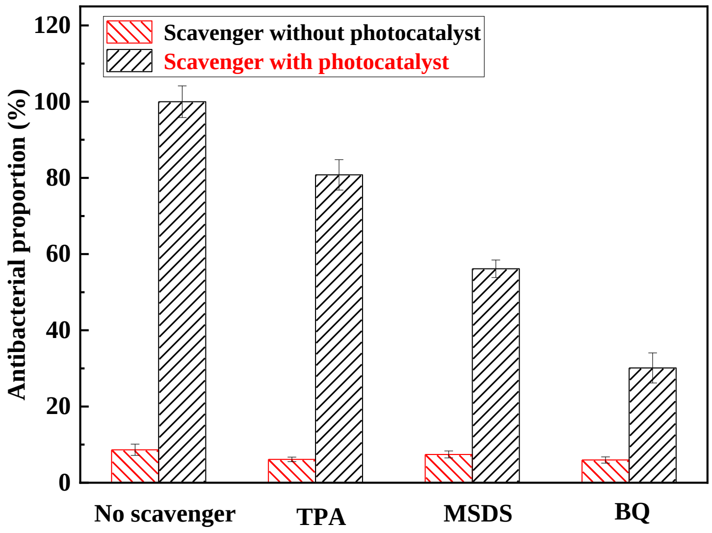

Figure S2. Photocatalytic antibacterial proportion after adding scavenger.

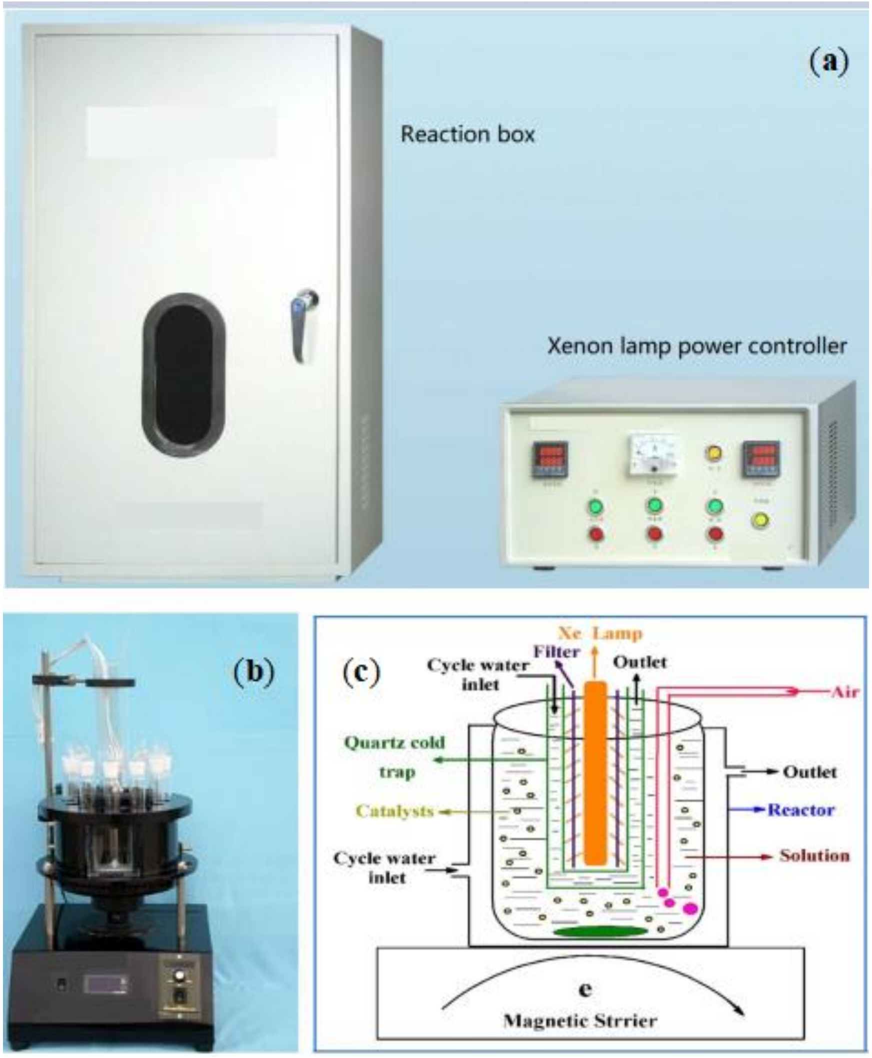

Figure S3. (**a**) Photocatalytic reaction instrument (**b**,**c**) Internal structure and principle of reaction box.
